# Melange

**Published:** 1885-10

**Authors:** 


### MELANGE.

A NEW SPECULUM.—Dr. R. T. Knox, of Gonzales, Texas, has converted Sims' Speculum, which he says he finds a clumsy and awkward thing to handle or carry (and we quite agree with him), into a very neat, useful and portable instrument. Of all the modifications of, and additions to Sims' Speculum we have seen, this is really about the only improvement. The principle of Sims' Speculum is good, and has immortalized Sims, but an improvement was needed, and Dr. Knox's fills the bill. It consists of reversing one blade, instead of having both turned in the same direction, and in hinging the handle in the middle. This converts it into something the shape of a capital S when open, and when closed—it shuts up like a knife—one blade fitting with the other " spoonfashion." In this shape it can be carried in the pocket. The principle objection to Sims' Speculum—unless we except its clumsy shape, which makes it a clumsy thing to carry—is that the free blade, when the instrument is in use, is in the way of the patient's clothing, whereas in this, it is turned away. The shank is rigged with quite an ingenious catch which braces it when open, and prevents its shutting up. The instrument is being manufactured by Tieman & Co., who report it popular. The improvement does not add to the cost of the speculum. It will be appreciated by all who see it.

ANOTHER GOOD MAN GONE.—As we go to press we are pained at the announcement in the Atlanta, Ga., *Medical and Surgical Journal*, of the death of DR. SAMUEL H. GRAY, of Barnesville, Ga. A Christian gentleman, a polished, cultivated physician, a devoted husband and father, and withal a useful citizen, his death, indeed, creates a void in every sphere of life. He was a brother of our esteemed cotemporary of the Atlanta *Medical and Surgical Journal*, Dr. James A. Gray, and of Rev. J. D. Gray, the presiding elder of the North Georgia Conference, to whom, and his bereaved family we tender our sincerest sympathy. *Requiescat in Pace.*

WITTY.—We suppose it is the same with jokes, as it is with fashions—the old ones come around and turn up at long intervals—somewhat like the Star of Bethlehem does. The *Columbus Medical Journal* is entitled to the credit of having brought forward, as a late thing, the most antiquated joke of which there is any record on earth. It is not exactly *ante*-deluvian, but rather *co-deluvian.* This sprightly journal gets off the fossil joke of the conversation between Noah and the "man who got left"—something about "it's not going to be much of a shower, anyway." Now, that is even more refreshing than a look at the new star, for the star has only been gone three hundred and fourteen years; while this excellent joke was in its dotage ere Sirius was evolved out of the nebulæ which, astronomers say, is stello-genetic. We imagine we see the rejuvinator of this side-splitting joke "shake his fat sides" like old Uncle Rat, in the nursery song, while he proceeds to "diagram" it (" Diagram a joke," *Columbus Medical Journal for August.*)

Now, "diagram a joke" *is* good; but we protest—the tendency is bad ; it will prove ruinous to the young and the feeble; and, after awhile, with such eminent authority for precedent, we will have—"parallelogram an idea;" and an endless variety of figures of speech—equally senseless—such, for instance, as "cheese it," which, no doubt, originated in some such silly way.

---

A BENEFIT—PASS HIM AROUND.—And now comes T. H. Bryant, "proprietor" "Waukesha Glenn," the "Queen of Mineral Waters," and poses as the *Prince of Advertising Frauds!* He is liberal with his ads.—five pages at a pop—but does not pay a cent. Beat us out of $26.66, the price of *five pages* one insertion; and we considered ourselves lucky to have been warned by two other leading journals worse stuck. He trades on wind as well as water. *This is free!*

---

FOR SALE.—An excellent country location, with a practice of $3000 per year. House and lot, drug store and 170 acres fine land. Will sell all, or residence alone. Address T. A. X., Wilderville, Falls county, Texas.

---

HYMENIAL.—Dr. E. T. Cook, of Willis, Tex., was married on 7th October (inst.) to Miss Minnie Thompson, daughter of J. S. Thompson, Sr., Esq., of same city.

We acknowledge the courtesy of cards, and beg to extend to our talented young friend our heartiest congratulations; and to both, our best wishes for a long life of happiness.

---

The captain of a Mexican steam ship tried to smuggle a case of yellow fever into New Orleans by deceiving the Quarantine officers, but the death of the man at Quarantine revealed the presence of the disease.

